# Assessment of Cardiac Energy Metabolism, Function, and Physiology in Patients With Heart Failure Taking Empagliflozin: The Randomized, Controlled EMPA-VISION Trial

**DOI:** 10.1161/CIRCULATIONAHA.122.062021

**Published:** 2023-04-18

**Authors:** Moritz J. Hundertmark, Amanda Adler, Charalambos Antoniades, Ruth Coleman, Julian L. Griffin, Rury R. Holman, Hanan Lamlum, Jisoo Lee, Daniel Massey, Jack J.J.J. Miller, Joanne E. Milton, Shveta Monga, Ferenc E. Mózes, Areesha Nazeer, Betty Raman, Oliver Rider, Christopher T. Rodgers, Ladislav Valkovič, Eleanor Wicks, Masliza Mahmod, Stefan Neubauer

**Affiliations:** 1 Oxford Centre for Clinical Magnetic Resonance Research (M.J.H., H.L., S.M., F.E.M., B.R., O.R., C.T.R., L.V., M.M., S.N.), Division of Cardiovascular Medicine, Radcliffe Department of Medicine, University of Oxford, John Radcliffe Hospital, UK.; 2 Acute Multidisciplinary Imaging and Interventional Centre (C.A., S.N.), Division of Cardiovascular Medicine, Radcliffe Department of Medicine, University of Oxford, John Radcliffe Hospital, UK.; 3 Department of Internal Medicine I, University Hospital Wuerzburg, Germany (M.J.H.).; 4 Diabetes Trials Unit, Oxford Centre for Diabetes, Endocrinology and Metabolism, Radcliffe Department of Medicine (A.A., R.C., R.R.H., J.E.M.), University of Oxford, UK.; 5 Department of Physics (J.M.), University of Oxford, UK.; 6 The Rowett Institute, University of Aberdeen, UK (A.N.).; 7 Oxford National Institutes of Health and Care Research Biomedical Research Centre, Oxford University Hospitals, Oxford, UK (R.R.H.).; 8 Boehringer Ingelheim International GmbH, Ingelheim, Germany (J.L.).; 9 Elderbrook Solutions GmbH on behalf of Boehringer Ingelheim Pharma GmbH and Co. KG, Biberach, Germany (D.M.).; 10 Wolfson Brain Imaging Centre, Department of Clinical Neurosciences, Cambridge Biomedical Campus, UK (C.T.R.).; 11 Department of Imaging Methods, Institute of Measurement Science, Slovak Academy of Sciences, Bratislava (L.V.).; 12 John Radcliffe Hospital, Oxford University Hospitals National Health Service Foundation Trust, UK (J.L., E.W.).; 13 Department of Clinical Medicine, Aarhus University, Denmark (J.J.M.).

**Keywords:** empagliflozin, heart failure, magnetic resonance spectroscopy, sodium-glucose transporter proteins

## Abstract

Sodium–glucose co-transporter 2 inhibitors (SGLT2i) have emerged as a paramount treatment for patients with heart failure (HF), irrespective of underlying reduced or preserved ejection fraction. However, a definite cardiac mechanism of action remains elusive. Derangements in myocardial energy metabolism are detectable in all HF phenotypes, and it was proposed that SGLT2i may improve energy production. The authors aimed to investigate whether treatment with empagliflozin leads to changes in myocardial energetics, serum metabolomics, and cardiorespiratory fitness.

**Methods:**

EMPA-VISION (Assessment of Cardiac Energy Metabolism, Function and Physiology in Patients With Heart Failure Taking Empagliflozin) is a prospective, randomized, double-blind, placebo-controlled, mechanistic trial that enrolled 72 symptomatic patients with chronic HF with reduced ejection fraction (HFrEF; n=36; left ventricular ejection fraction ≤40%; New York Heart Association class ≥II; NT-proBNP [N-terminal pro-B-type natriuretic peptide] ≥125 pg/mL) and HF with preserved ejection fraction (HFpEF; n=36; left ventricular ejection fraction ≥50%; New York Heart Association class ≥II; NT-proBNP ≥125 pg/mL). Patients were stratified into respective cohorts (HFrEF versus HFpEF) and randomly assigned to empagliflozin (10 mg; n=35: 17 HFrEF and 18 HFpEF) or placebo (n=37: 19 HFrEF and 18 HFpEF) once daily for 12 weeks. The primary end point was a change in the cardiac phosphocreatine:ATP ratio (PCr/ATP) from baseline to week 12, determined by phosphorus magnetic resonance spectroscopy at rest and during peak dobutamine stress (65% of age-maximum heart rate). Mass spectrometry on a targeted set of 19 metabolites was performed at baseline and after treatment. Other exploratory end points were investigated.

**Results:**

Empagliflozin treatment did not change cardiac energetics (ie, PCr/ATP) at rest in HFrEF (adjusted mean treatment difference [empagliflozin – placebo], –0.25 [95% CI, –0.58 to 0.09]; *P*=0.14) or HFpEF (adjusted mean treatment difference, –0.16 [95% CI, –0.60 to 0.29]; *P*=0.47]. Likewise, there were no changes in PCr/ATP during dobutamine stress in HFrEF (adjusted mean treatment difference, –0.13 [95% CI, –0.35 to 0.09]; *P*=0.23) or HFpEF (adjusted mean treatment difference, –0.22 [95% CI, –0.66 to 0.23]; *P*=0.32). No changes in serum metabolomics or levels of circulating ketone bodies were observed.

**Conclusions:**

In patients with either HFrEF or HFpEF, treatment with 10 mg of empagliflozin once daily for 12 weeks did not improve cardiac energetics or change circulating serum metabolites associated with energy metabolism when compared with placebo. Based on our results, it is unlikely that enhancing cardiac energy metabolism mediates the beneficial effects of SGLT2i in HF.

**Registration:**

URL: https://www.clinicaltrials.gov; Unique identifier: NCT03332212.

**Clinical Perspective**

**What Is New?**

- Based on preclinical results, it has been suggested that sodium–glucose co-transporter 2 inhibitor (SGLT2i) treatment may lead to a myocardial substrate switch and thus, improve cardiac energy reserve.
- This mechanistic cardiac magnetic resonance spectroscopy and imaging trial investigated 72 patients with symptomatic, nonischemic heart failure (36 with heart failure with reduced ejection fraction 36 with heart failure with preserved ejection fraction) and assessed measures of cardiac energy metabolism, myocardial function, and structure.
- Compared with placebo, treatment with the SGLT2i empagliflozin for 12 weeks did not enhance myocardial energy metabolism or serum metabolites associated with energy metabolism.

**What Are the Clinical Implications?**

- SGLT2 inhibition with empagliflozin (10 mg once daily) for 3 months did not improve cardiac energetics in patients with heart failure with reduced ejection fraction or heart failure with preserved ejection fraction.
- Thus, the proposed “thrifty fuel hypothesis,” suggesting improved energy provision to be a central mechanism underlying the SGLT2i beneficial clinical effects observed in patients with heart failure, could not be confirmed.
- Our study suggests that effects other than enhanced energy metabolism may explain the favorable effects of empagliflozin observed in patients with heart failure.

Heart failure (HF), a clinically complex multiorgan syndrome, has a plethora of possible causes, and despite multiple treatment innovations, overall prognosis remains poor.^1^ Improvements in survival after acute ischemic events have led to a constantly rising HF prevalence, particularly in an aging population.^1^ Consequentially, recurrent HF hospitalizations in combination with increasing morbidity and high prevalence of comorbidities add to the heavy burden for patients and health care systems. With projections of HF prevalence affecting up to 9% of all Americans ≥65 years of age within the next few years, innovative treatments addressing this ongoing epidemic are urgently required.^2,3^

After their serendipitous discovery as novel heart failure drugs,^4–7^ sodium–glucose co-transporter 2 inhibitors (SGLT2i) have emerged as a cornerstone of treatment for both HF with reduced ejection fraction (HFrEF) and HF with preserved ejection fraction (HFpEF), as well as other cardiac conditions.^8–10^ Nevertheless, it remains unclear how a drug that inhibits transport proteins not expressed on cardiomyocytes^11^ may lead to substantially reduced risk of hospitalization for HF or cardiovascular death shortly after treatment onset.

SGLT2i induce glucosuria and, thus, lead to a caloric deficit of approximately 250 calories per day.^12^ As a compensatory measure, this increases free fatty acid oxidation, and, depending on the underlying condition, levels of beta-hydroxybutyrate (β-OHB), an energy source used by cardiomyocytes.^13^ As a result, the “thrifty substrate hypothesis”^14^ was put forward connecting the salutary effects in HF subgroups of EMPA-REG OUTCOME to a shift in cardiac energy substrates. Subsequently, possible energetic effects were explored using preclinical HF models with limited generalizability (in vitro experiments or animal models)^15–17^; inconsistent results of efficacy are noted across these studies.^18,19^

Irrespective of the underlying etiology, HF displays a reduction in myocardial energy provision, affecting all components of the energetic system.^20^ Phosphocreatine levels, the main energetic buffer in the heart, decline substantially earlier and comparatively more than ATP, making the ratio of PCr and ATP (PCr/ATP) a sensitive marker of the overall energetic state of the heart.^21–23^ In HFrEF, PCr/ATP correlates with New York Heart Association class and ejection fraction, and in HFpEF, PCr/ATP correlates with severity of diastolic impairment.^24–27^

No previous study has investigated the metabolic effects of SGLT2i in patients with HFrEF or HFpEF in vivo. Accordingly, EMPA-VISION (Assessment of Cardiac Energy Metabolism, Function and Physiology in Patients With Heart Failure Taking Empagliflozin) is the first prospective, randomized, double-blind, placebo-controlled trial assessing the effects of empagliflozin treatment on cardiac energetics and physiology.

## Methods

The trial design and methods of EMPA-VISION (URL: https://www.clinicaltrials.gov; Unique identifier: NCT03332212; EudraCT-Number: 2017-000376-28) have been published separately.^28^ The trial, its protocol, and further amendments to it were approved by the South Central–Oxford C Research Ethics Committee Health Research Authority and the Medicines Healthcare Regulatory Agency. All patients, whether enrolled or not, provided written informed consent, and investigations were undertaken in accordance with institutional policies of the Declaration of Helsinki. Applications to provide the data supporting the findings of this article will be available from the corresponding author upon reasonable request.

### Patients

EMPA-VISION comprised 2 separate cohorts with HFrEF (left ventricular ejection fraction [LVEF] ≤40%) and HFpEF (LVEF ≥50%). Trial patients were considered eligible with an established diagnosis of nonischemic, chronic HF with typical signs (NT-proBNP [N-terminal pro-B-type natriuretic peptide] >125 pg/mL in sinus rhythm or >600 pg/mL in atrial fibrillation) and symptoms (New York Heart Association classes II–IV) and appropriate doses of guideline-directed HF medical therapy. In addition, patients with HFpEF were required to display significant signs of adverse structural remodeling (left atrial volume index >34 mL/m^2^; left ventricular mass index >95 g/m^2^ [women] and >115 g/m^2^ [men]). All patients underwent computed tomography coronary angiography to exclude significant, flow-limiting coronary artery disease (luminal stenosis >60%) or an ischemic HF etiology. Patients with significant coronary artery disease, ischemia, implanted devices, recent (within 1 week before screening visit) decompensated HF, or severely impaired renal function (creatinine clearance <30 mL/min by Cockcroft–Gault formula) were excluded. A comprehensive list of inclusion and exclusion criteria are provided in Table S1. To standardize metabolic investigations as much as possible, all patients were assessed in a fasting state (>6 hours before the visit), and visits at baseline and week 12 were conducted at the same time of day with an identical order of assessments.

### Randomization Process

**Figure 1.**
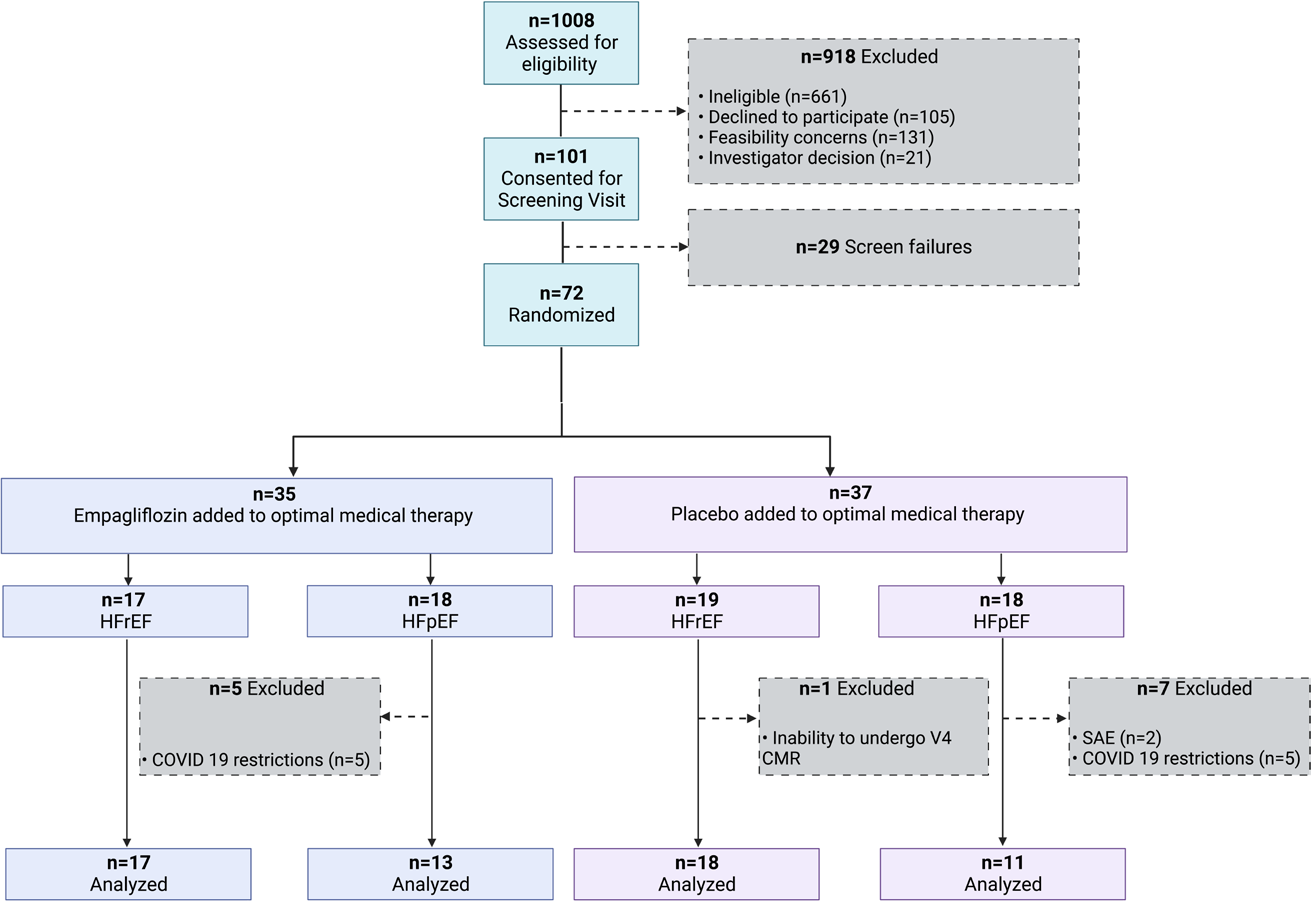
**Patient flow diagram for the EMPA-VISION double-blind, phase-III randomized controlled trial.** Patients (n=1008) were assessed for eligibility; 101 patients were consented and underwent a screening visit. Of those, 72 were eventually eligible and randomly allocated to either 10 mg of empagliflozin (n=35) or matching placebo (n=37) once daily, stratified in their respective cohorts (HFrEF and HFpEF). One participant with HFrEF in the placebo group withdrew from further participation before the individual end of treatment (visit 4). In the HFpEF cohort, 5 patients in the empagliflozin arm and 5 in the placebo arm were excluded due to missing data because of COVID-19 lockdown restrictions. Two patients in the placebo group withdrew from treatment due to serious adverse events. CMR indicates cardiovascular magnetic resonance; HFpEF, heart failure with preserved ejection fraction; HFrEF, heart failure with reduced ejection fraction; and SAE, serious adverse event.

During their baseline visit (visit 2), eligible patients were randomly assigned to receive either 10 mg of empagliflozin or matching placebo once daily in a 1:1 fashion (Figure 1). The drug assignment was carried out using an interactive web response system, and the randomization list involved a pseudorandom number generator ensuring the resulting treatment was both reproducible and nonpredictable with a block size of 4.

### Study Visit Schedule

Patients invited to participate attended for a total of 4 visits (Table S2): (1) screening (visit 1: days −21 to 0); (2) randomization (visit 2: day 1); (3) safety (visit 3: day 15±1); and (4) end of treatment (visit 4: day 84±4). A follow-up phone call (visit 5: days 7–14 after visit 4) was conducted for safety purposes. Table S2 details a full study flowchart including investigations on each respective visit.

### Study Treatment

The study drug (empagliflozin [10 mg]) and matching placebo (one tablet) were commenced on site once daily in the investigator’s presence on the day of the randomization visit (visit 2). Treatment compliance was assessed by staff during all follow-up visits, and safety monitoring of blood pressure and urine assessment for urinary tract or genital infections were performed. All patients and staff involved in the conduct of the trial were blinded to the assigned treatments.

### Primary End Point

The primary end point defined in this trial was the change in cardiac PCr/ATP from baseline to week 12, measured by phosphorus-31 (^31^P) magnetic resonance spectroscopy (MRS).

### Secondary End Points

No secondary end points were defined for this trial.

### Exploratory End Points

A variety of exploratory end points were defined in the trial protocol as well as the trial statistical analysis plan. These included measures of energy metabolism at rest and during dobutamine stress, assessment of myocardial triglyceride content (MTG) by proton MRS, cardiac function and volumes at rest and during dobutamine stress, measures of cardiac fibrosis (late gadolinium enhancement, extracellular volume, and shortened modified look-locker inversion recovery [ShMOLLI]) T1, as well as blood biomarkers relating to drug effects on metabolism or neurohormonal activation. A full list of exploratory outcomes is provided in the trial statistical analysis plan in the Supplemental Material.

### Safety Evaluation

Safety parameters included adverse events, predefined adverse events of special interest (eg, hepatic injury, decreased renal function, and diabetic ketoacidosis), and specific adverse events defined for this study (eg, hypoglycemic events, genital infections, acute pyelonephritis, sepsis, urinary tract infections, bone fractures, hepatic injury, ketoacidosis, and acute kidney injury), clinical safety laboratory assessments, vital signs, 12-lead ECG, and New York Heart Association class. Details on collection of adverse events and overall safety of empagliflozin in patients with HFrEF and HFpEF can be found in Table S3.

### Cardiovascular Magnetic Resonance

All cardiovascular magnetic resonance (CMR) assessments took place in the Oxford Centre for Clinical Magnetic Resonance Research (OCMR, University of Oxford). ECG-gated magnetic resonance imaging was performed at baseline (visit 2) and after 12 weeks of treatment using a 3-T scanner (MAGNETOM Prisma; Siemens Healthineers, Erlangen, Germany). Imaging sequences included steady-state free precession cine imaging, ShMOLLI T1–mapping, resting perfusion and late gadolinium enhancement imaging. A detailed description of the entire scanning protocol is provided in our published design manuscript.^28^ All image analyses were provided by our in-house imaging core laboratory with analysts blinded to treatment status. Before all analyses, each individual data set was assessed for quality (from 0 [indicating OK] to 3 [indicating data missing or not analyzable]) and cross-checked by 2 independent analysts. All outputs were reanalyzed before finalization of the output with monitoring and cross-checking of results provided by the head of the Corelab.

Binuclear (^31^P and ^1^H) MRS was performed at rest using a 3-T magnetic resonance scanner (MAGNETOM Trio; Siemens Healthineers). Participants were positioned prone over the center of a 3-element dual-tuned ^1^H/^31^P surface coil in the isocenter of the magnetic resonance scanner. A nongated, 3D acquisition–weighted, ultra-short echo-time chemical shift imaging sequence was used with saturation bands placed over liver diaphragm and skeletal muscle, as previously described.^29^ All analyses were performed by an experienced investigator (M.J.H.; >5 years of CMR experience) and consisted of a semiautomated data-quantification pipeline using the OXSA toolbox within a MATLAB (Matrix Laboratory) implementation of the advanced method for accurate, robust, and efficient spectral fitting MRS spectral fitting algorithm.^30^ This process recombined and quantified the raw data before correction of the acquired PCr and ATP signals for partial saturation by using literature values of T1 and then calculated the PCr/ATP ratio, which was expressed as average PCr/ATP. For consistency reasons, the reported PCr/ATP was taken from the interventricular septum of the third basal cardiac short axis slice below the left ventricular outflow tract. All analyses were repeated by a blinded expert (L.V.; 9 years of CMR experience).

MTG of the heart was assessed via proton MRS using an 18-channel surface coil supine in end-diastole and expiration. This enabled acquisition of water-suppressed, and non–water-suppressed lipid spectra, allowing the calculation of MTG.

### Cardiorespiratory Fitness

Assessment of cardiorespiratory fitness was performed via cardiopulmonary exercise testing (CPET). Patients were seated on a stationary exercise bike (Ergoline GmbH, Bitz, Germany) for resting spirometry followed by CPET using breath-by-breath respiratory gas analysis (Metalyzer 3B, Cortex Biophysik, Leipzig, Germany). A standardized incremental exercise protocol was used, and patients were encouraged to exercise until at least a respiratory exchange ratio ≥1.1 was reached. Oxygen saturation, capillary lactate, and subjective exertion (Borg Scale) were assessed every 2 minutes, and peak oxygen consumption (V ˙ o_2_) was measured at maximal exhaustion.

### Bloods and Biomarkers

Patients were fasting for at least 6 hours before venous blood samples were drawn. These were processed in-house, snap-frozen (−20º or −80ºC), and then shipped to a central laboratory (Labcorp, Geneva, Switzerland) for biomarker analysis or to an academic collaborator (Julian Griffin, professor, Imperial College London, UK) for a targeted metabolomic analysis 19 serum metabolites relating to energy metabolism.^31^

### Statistical Analysis

Statistical analyses of efficacy data were conducted using SAS 9.4 (SAS Institute, Cary, NC) at the Diabetes Trials Unit (Oxford Centre for Diabetes, Endocrinology and Metabolism, University of Oxford, UK) after a prespecified statistical analysis plan (Supplemental Material). Due to the lack of specific data describing the impact of SGLT2i on measures of energy metabolism in patients with HF, the sample size was estimated using results of a previous trial investigating a metabolic modulator that increased PCr/ATP in a cohort of 25 nonischemic HFrEF patients with a treatment difference of 0.37.^32^ Consequently, detecting a treatment difference of 0.3 (SD, 0.28) with β=0.8, and a 2-sided significance level of 0.05, the sample size required was determined to be n=30 participants per cohort. Allowing for a maximum dropout rate of 30%, we anticipated recruitment of a maximum possible number of 43 patients per cohort (86 patients total).

The primary end point analysis was performed on the per protocol set (PPS) of patients with valid PCr/ATP measurements available at baseline and week 12. The formal analysis employed an analysis of variance model for the primary end point hypothesis testing. Response (ie, outcome) was defined as the change (PCr/ATP absolute change) from baseline to week 12 calculated for each patient by subtracting the baseline PCr/ATP measurement from the 12-week measurement and was adjusted for treatment (empagliflozin versus placebo), history of type 2 diabetes (yes or no), and history of atrial fibrillation (yes or no). A sensitivity analysis of the primary end point was introduced that included baseline PCr/ATP value as a covariate in an analysis of covariance model. Furthermore, the same ANCOVA model was employed on the randomized set (in line with the intention-to-treat principle) of patients to check for an unbiased estimation of the primary end point result.

Subgroup analyses were performed to assess the homogeneity of treatment effects on changes in the PCr/ATP ratio in different subgroups (eGFR [<60 versus >60 mL/(min1.73 m^2^)], diabetes mellitus [yes or no], and atrial fibrillation [yes or no] subgroups). The same ANOVA model as used for the primary end point was employed with the addition of subgroup term (if not already fitted) and the treatment by subgroup interaction term.

The analysis of exploratory end points was performed with available data using descriptive statistics or ANCOVA with no adjustment for multiple testing. All summaries were produced for both cohorts using all available data.

For analysis of the metabolomic data, a Wilcoxon rank-sum test was applied. The metabolites were then sorted by the signed significance of each respective test. Signed significance is defined as the negative of the logarithm (base 10) of the test probability value and multiplied by the sign of the median (the difference in medians between the 2 samples). A conventional principal component analysis (ie, using singular value decomposition) was computed by the *pcaMethods R package*^33^ (version 1.84) with mean centering and scaling. There were no missing data, and 5 components were computed.

### Data Sharing Statement

To ensure independent interpretation of clinical study results and enable authors to fulfill their role and obligations under the International Committee of Medical Journal Editors criteria, Boehringer Ingelheim grants all external authors access to clinical study data pertinent to the development of the publication. In adherence with the Boehringer Ingelheim Policy on Transparency and Publication of Clinical Study Data, scientific and medical researchers can request access to clinical study data after publication of the primary article in a peer-reviewed journal, regulatory activities are complete, and other criteria are met. Researchers should use the link to request access to study data and visit https://www.mystudywindow.com/msw/datasharing for further information.

## Results

Patient recruitment took place at a single center (OCMR, Oxford, UK) from March 2018 to May 2020. A total of 101 patients performed a screening visit, and 72 patients were eventually enrolled and randomly assigned treatment. Of those, 36 patients were in the HFrEF cohort (19 placebo and 17 empagliflozin) and 36 in the HFpEF cohort (18 placebo and 18 empagliflozin). One patient who was randomized into the placebo arm of the study was excluded from data analysis of the PPS due to premature end of follow-up. The HFrEF cohort of patients was recruited and followed up in full before recruitment in the HFpEF cohort finished. Thus, because of the unprecedented global COVID-19 pandemic and far-reaching effects of national lockdowns, all face-to-face research activities were suspended from March 24, 2020, onward. This inability to conduct end-of-treatment visits after 12 weeks of treatment led to an exclusion of 13 (36.1%) patients from the randomized set and a subsequent numeric reduction of the PPS to 24 patients (13 on empagliflozin and 11 on placebo) in the HFpEF cohort.

### Baseline Characteristics

A summary of the baseline characteristics of patients randomized to treatment is presented in Table 1.

**Table 1.**
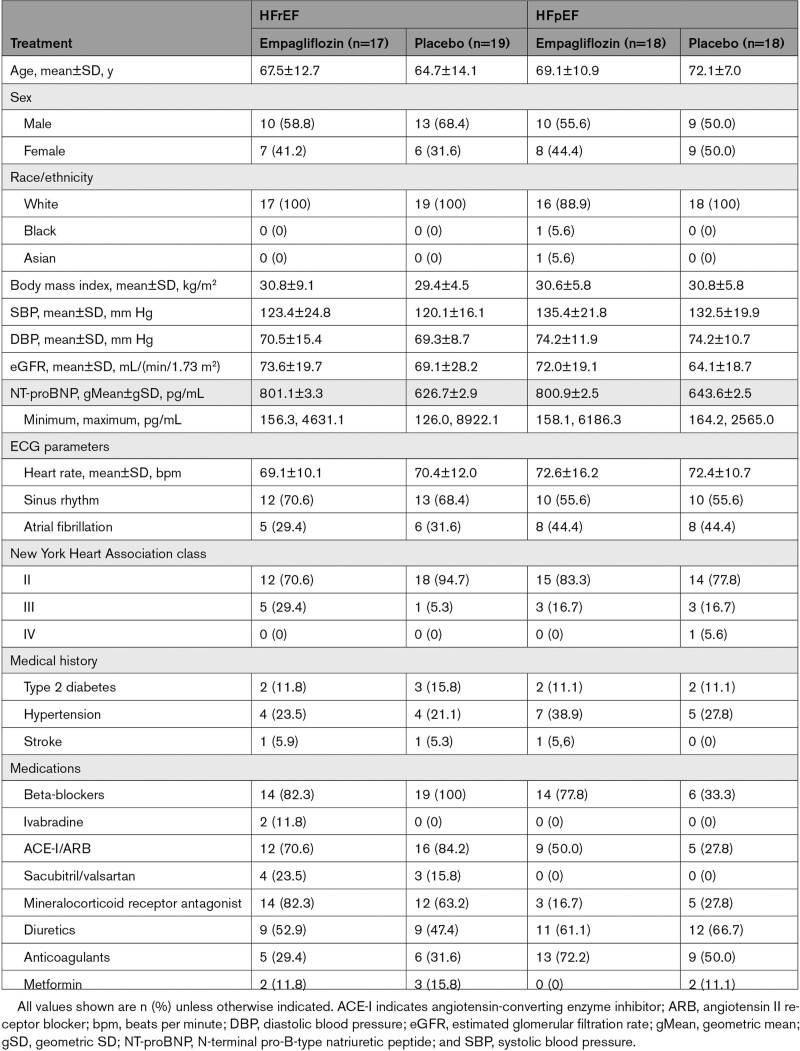
Baseline Characteristics of the Randomized Participants

The mean duration of exposure to treatment was 85.3 days (SD, 2.4) for the empagliflozin group (n=35) and 84.55 days (SD, 3.11) for the placebo group (n=36).

Overall, 58.3% of patients were men, nearly all were White (97.2%), 1.4% were Black, 1.4% were of Asian ancestry, and the mean age was 68.33 years (SD, 11.51). In the HFrEF cohort, all patients had an LVEF ≤40% before treatment. The mean eGFR was 73.6 mL/(min/1.73 m^2^) (SD, 19.7) for the empagliflozin group and 69.1 mL/(min/1.73 m^2^) (SD, 28.2) for the placebo group, and the geometric mean NT-proBNP was 801.1 pg/mL (gSD, 3.30) and 626.7 pg/mL (gSD, 2.90) for the empagliflozin and placebo group, respectively.

In the HFpEF cohort, all patients had an LVEF of ≥50%. Mean eGFR was 72.0 mL/(min/1.73 m^2^) (SD, 19.1) for the empagliflozin group and 64.1 mL/(min/1.73 m^2^) (SD, 18.7) for the placebo group, with a geometric mean NT-proBNP of 800.90 pg/mL (gSD, 2.48) and 643.60 (gSD, 2.50) in the empagliflozin and placebo groups, respectively.

It was anticipated that patients with type 2 diabetes would account for 30% to 40% of those randomized; however, only 12.55% of all patients enrolled eventually had concomitant type 2 diabetes.

### Primary End Point: Cardiac Energetics (PCr/ATP Ratio)

**Figure 2.**
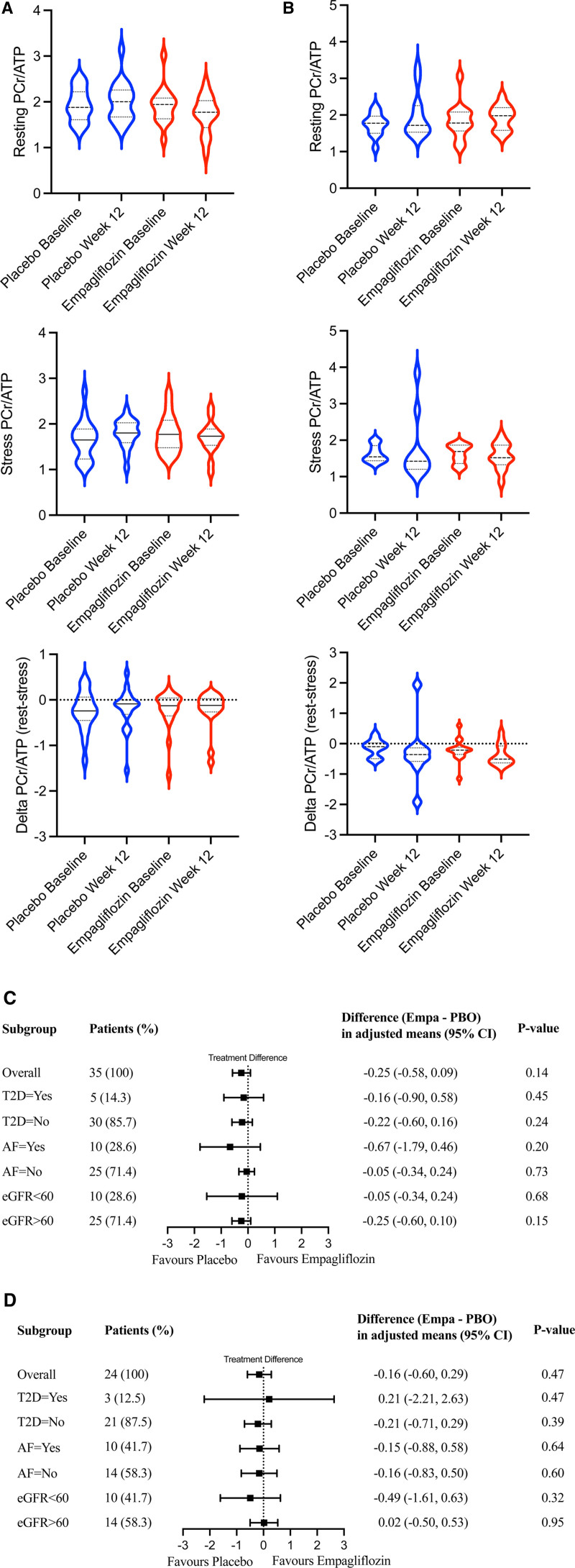
Cardiac energetics (PCr/ATP). Cardiac energetics (PCr/ATP) defined by phosphorus-31 (^31^P) magnetic resonance spectroscopy violin plots (including median and interquartile range) for the placebo and the empagliflozin treatment groups at baseline and 12 weeks after the respective treatment. PCr/ATP remained unchanged in both HFrEF (**A**) and HFpEF (**B**) after 12 weeks of empagliflozin treatment at rest (top row) and during dobutamine stress (middle row), with 65% of age-maximum heart rate (ie, 220-age). Furthermore, the difference of PCr/ATP at rest minus dobutamine stress (ΔPCr/ATP) from baseline to week 12 was equally unchanged (bottom row). Subgroup analyses in HFrEF (**C**) and HFpEF (**D**), including the overall presence or absence of T2D, AF, and eGFR, were consistent with the overall neutral results. AF indicates atrial fibrillation; eGFR, estimated glomerular filtration rate; Empa, empagliflozin; HFpEF, heart failure with preserved ejection fraction; HFrEF, heart failure with reduced ejection fraction; PBO, placebo; and PCr/ATP, phosphocreatine/ATP ratio.

After 12 weeks of treatment with 10 mg of empagliflozin, there was no significant difference regarding the change in the resting PCr/ATP compared with placebo (Table 2). In the HFrEF cohort, the mean change was −0.18 (SE, 0.12) for empagliflozin versus 0.07 (SE, 0.11) for placebo, with an adjusted mean treatment difference of −0.25 (SE, 0.16 [95% CI, −0.60 to 0.10]; *P*=0.14). In the HFpEF cohort, there was no significant difference between empagliflozin and placebo regarding the change in resting PCr/ATP from baseline to week 12, with an adjusted mean change of 0.10 (SE, 0.14) for empagliflozin versus 0.26 (SE, 0.16) for placebo. The adjusted mean treatment difference was −0.16 (SE, 0.21 [95% CI, −0.60 to 0.29]; *P*=0.47). These results were consistent with sensitivity analyses repeating the ANOVA on the intention-to-treat population of patients, as well as including baseline PCr/ATP as a covariate using an ANCOVA for the PPS of patients. Subgroup analyses considering kidney disease (eGFR <60 mL/[min/1.73 m^2^] versus >60 mL/[min/1.73 m^2^]), diabetes status (type 2 diabetes: yes or no), and presence of atrial fibrillation (yes or no) were consistent with the primary analysis in both cohorts (Figure 2C and 2D).

**Table 2.**
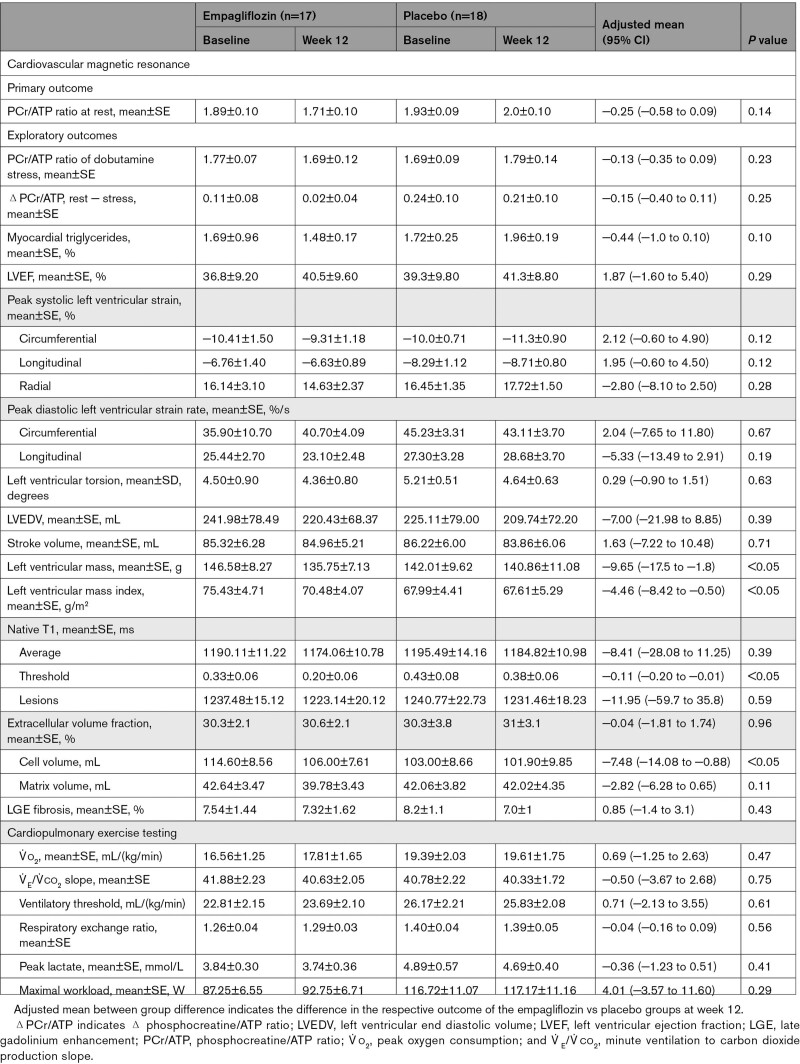
**Changes in Primary and Exploratory Outcomes With Empagliflozin or Placebo (Baseline to Week 12): Heart Failure With Reduced Ejection Fraction (Per Protocol Set**)

### Exploratory End Points

#### Dobutamine Stress PCr/ATP Ratio

As expected, PCr/ATP during dobutamine stress (65% age-maximum heart rate) was reduced compared with resting conditions (Table 2 A and B). However, empagliflozin treatment did not result in an improvement of energetics under dobutamine infusion (treatment difference for the stress PCr/ATP [empagliflozin – placebo, baseline to week 12] for patients with HFrEF, −0.13 (SE, 0.11 [95% CI, −0.40 to 0.10]; *P*=0.23); HFpEF, −0.22 (SE, 0.21 [95% CI, −0.66 to 0.23]); *P*=0.32). Likewise, no change was observed in the difference from rest to stress (ΔPCr/ATP), after 12 weeks of treatment, in either HFrEF (adjusted mean treatment difference, −0.15 [SE, 0.12 (95% CI, −0.40 to 0.11)]; *P*=0.25) or HFpEF (adjusted mean treatment difference, −0.07 [SE, 0.28 (95% CI, −0.51 to 0.66)]; *P*=0.80)]. Absolute changes (week 12 – baseline) in resting and dobutamine stress PCr/ATP are provided in Figure S1.

### Serum Metabolomics

**Figure 3.**
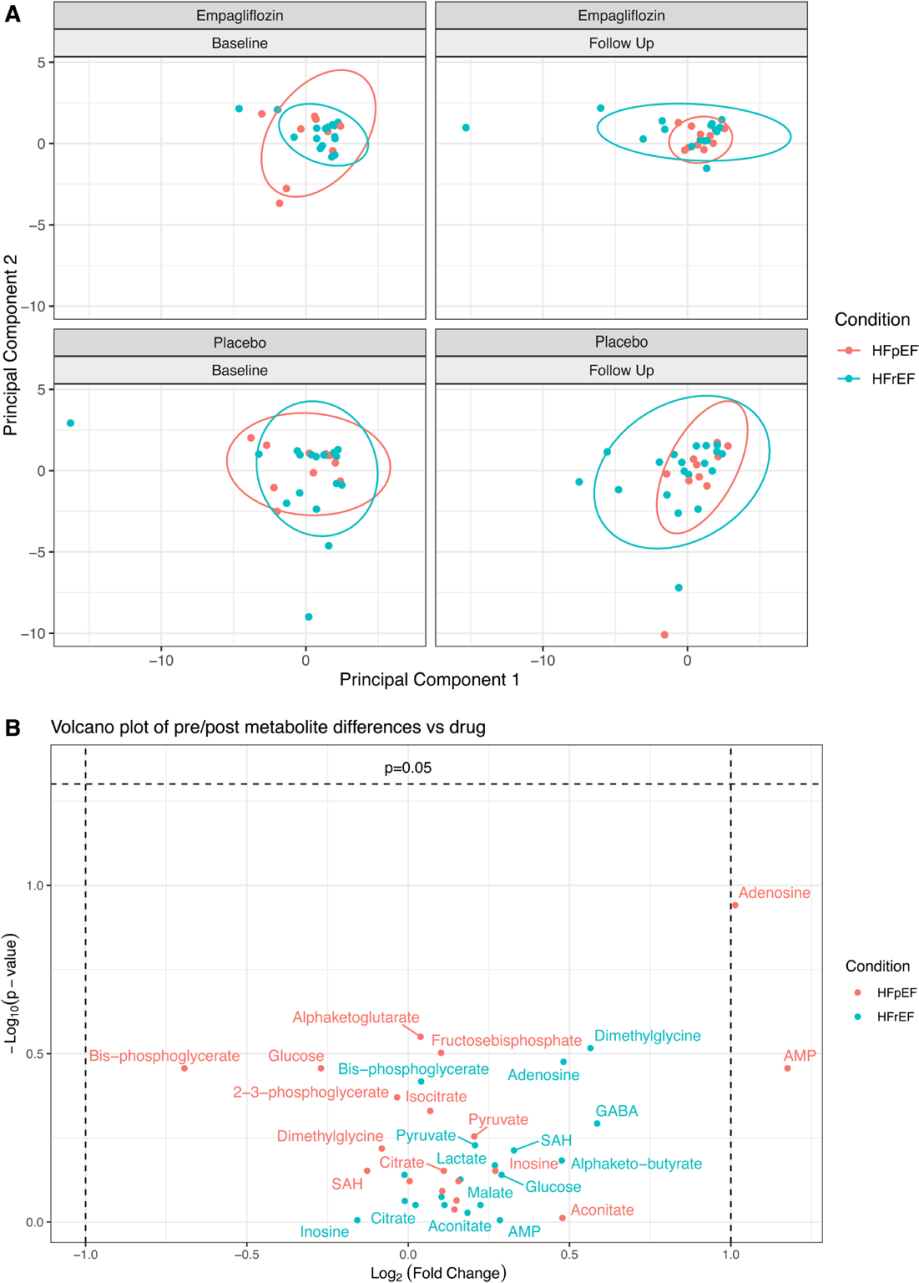
Targeted serum metabolomics. **A**, Principal component analysis showed no significant differences in clustering Euclidean distance by either condition (HFpEF or HFrEF; *P*=0.23) or group (empagliflozin vs placebo; *P*=0.38). **Top** (right and left), empagliflozin. **Bottom** (right and left), placebo. Red indicates HFpEF; blue indicates HFrEF. **B**, Volcano plot visualizing the degree of statistical significance (Wilcoxon rank-sum test) on the *y* axis vs the magnitude of change (fold change of medians) on the *x* axis, with a *P* value <0.05 considered statistically significant. Data points represent the difference of treatment from baseline to week 12 (between empagliflozin and placebo) in the HFpEF and HFrEF groups, respectively. HFpEF indicates heart failure with preserved ejection fraction; HFrEF, heart failure with reduced ejection fraction; GABA, γ-aminobutyric acid; and SAH, S-adenosyl-L-homocysteine.

The effects of empagliflozin and placebo treatment on a set of 19 targeted metabolites were investigated with a principal component analysis of serum metabolomic samples (Figure 3A). No change induced by treatment with empagliflozin versus placebo could be observed in HFrEF and HFpEF (Figure 3B for statistical significance versus magnitude of change).

### CMR Imaging

In the HFrEF cohort, changes regarding left ventricular mass and mass index after empagliflozin treatment were observed: left ventricular mass reduction (adjusted mean treatment difference) was −9.65 g (SE, 3.83 [95% CI, −17.49 to −1.81]; *P*=0.02), which was consistent when mass was indexed to body surface area (left ventricular mass index, −4.46 g/m^2^ [SE, 1.94 (95% CI, −8.42 to −0.50)]; *P*=0.03).

The changes in ShMOLLI T1–derived measures of myocardial tissue characterization (native, lesions, and threshold) were numerically greater in the empagliflozin group with the threshold T1 reaching nominal statistical significance (Table 2). Although there was no change in extracellular volume when deriving left ventricular cellular and matrix volumes separately, as previously described,^34^ the change in cellular volume in the empagliflozin group (−8.6 mL ;SE, 2.00) compared with placebo (−1.1 mL [SE, 2.47]; Table 2) reached significance, whereas the reduction in left ventricular matrix volume did not (*P*=0.10).

For patients with HFpEF (Table 3), there were no nominally significant changes from baseline to week 12 (empagliflozin – placebo) regarding left ventricular mass, volumes, or function.

**Table 3.**
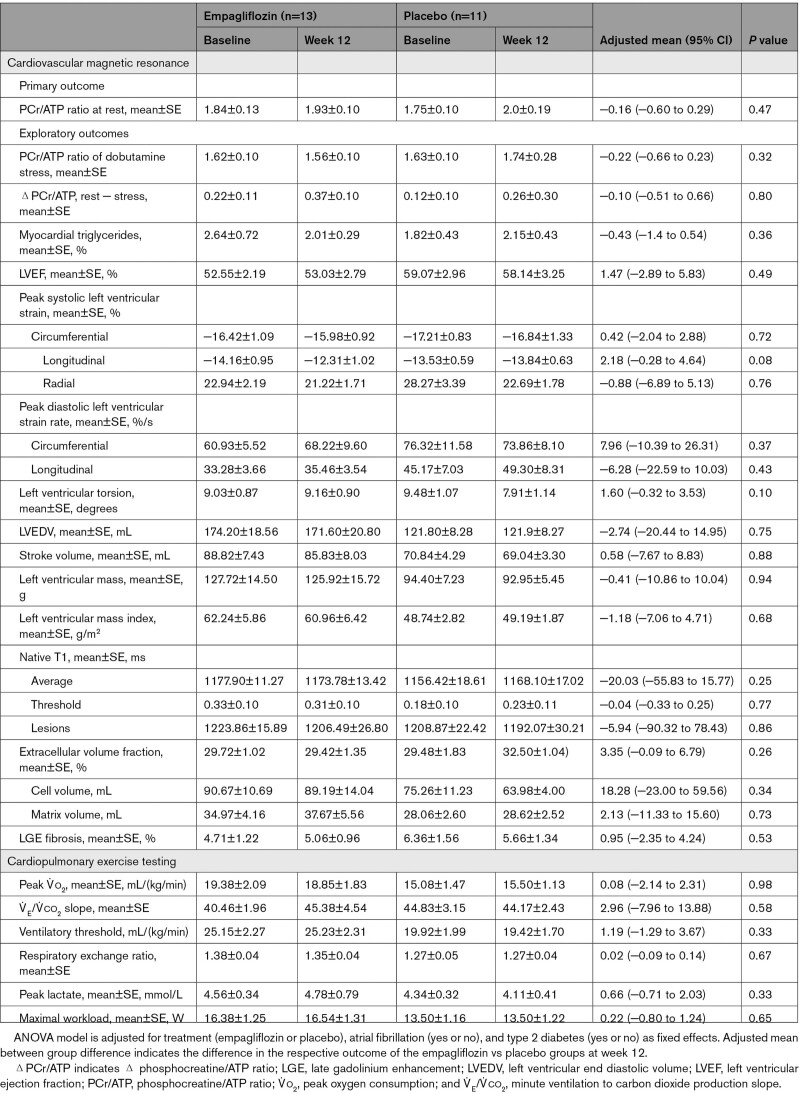
**Changes in Primary and Exploratory Outcomes With Empagliflozin or Placebo (Baseline to Week 12): Heart Failure With Preserved Ejection Fraction (Per Protocol Set**)

We observed a trend for an improvement in peak systolic longitudinal strain with empagliflozin (adjusted mean treatment difference, 2.18% [SE, 1.16 (95% CI, −0.28 to 4.64)]; *P*=0.08) and torsion (adjusted mean treatment difference, 1.60° [SE, 0.91 (95% CI, −0.32 to 3.53)]; *P*=0.10).

### Myocardial Triglyceride Content

Empagliflozin treatment led to a trend in decrease of MTG, measured via proton MRS, comparable in patients with HFrEF (adjusted mean treatment difference, −0.44% [95% CI, −0.97 to 0.08]; *P*=0.10) and HFpEF (adjusted mean treatment difference, −0.43% [95% CI, −1.39 to 0.54]; *P*=0.60)], although this did not meet nominal statistical significance.

### Serum Biomarkers

For most serum-derived biomarkers, there were no significant differences from baseline to week 12 between the empagliflozin and placebo group (Figure S2). Likewise, concentrations of the ketone body β-OHB and the amount of circulating free fatty acids did not change after treatment with empagliflozin.

### Cardiorespiratory Fitness and Quality of Life

Overall, we did not detect significant changes in exercise-derived measures after CPET (Tables 2 and 3) in HFrEF or HFpEF. Quality of life, assessed by Kansas City Cardiomyopathy Questionnaire, showed a trend toward greater improvements with empagliflozin (mean change in overall summary score, 9.81±1.27) compared with placebo (4.24±1.39).

### Safety

Details on adverse events stratified by treatment group can be found in Table S3. Overall, treatment with empagliflozin was safe and well tolerated, with more adverse events in the placebo group (n=19) than the empagliflozin group (n=17). Adverse events severity was equally distributed between mild (50%) and moderate (50%) in both trial arms. A total of 8 serious adverse events (placebo group, n=7; empagliflozin group, n=1) were recorded.

## Discussion

The impact of SGLT2i treatment on myocardial energy metabolism in patients with HF remains hotly debated but continues to be poorly understood. EMPA-VISION is the first trial to investigate effects of empagliflozin treatment in patients with HFrEF and HFpEF on cardiac energetics and metabolism. The principal findings of our trial are: (1) after 12 weeks of empagliflozin (10 mg once daily) versus placebo, there was no difference in the primary end point (myocardial energetics in vivo via ^31^P MRS [PCr/ATP at rest]), in patients with HFrEF nor with HFpEF (likewise, no treatment effects could be demonstrated during dobutamine stress assessment of PCr/ATP); and (2) in keeping with the neutral effects on cardiac energetics, we did not observe significant changes in a targeted metabolomic assay investigating 19 serum metabolites related to energy metabolism in either cohort.

### Thrifty Substrate Hypothesis of SGLT2i

SGLT2i induce renal glucosuria, which, in turn, lowers the insulin-to-glucagon ratio and stimulates lipolysis, prompting a mild increase in hepatic production of ketone bodies.^35^ It has been described that the failing heart uses ketone bodies for energy production linearly to their availability.^13^ Ketones have been touted as myocardial superfuels and are assumed to improve myocardial energy efficiency with short-term β-OHB infusion showing to increase cardiac function and output in patients with HFrEF.^13,14,36^ As such, it was hypothesized that the beneficial clinical effects of SGLT2i might (partially) be attributed to enhanced myocardial energetics.^14^ Overall, data on changes in substrate utilization and energy metabolism in HF after SGLT2i treatment are scarce, and results are conflicting.^15,17,19,37^ Human data are limited to small studies in diabetic patients without HF, with a randomized controlled trial (n=56) showing no difference in energetics (secondary end point), whereas a longitudinal study (n=28) did find an improvement.^38,39^

### Myocardial Energetics and SGLT2i in HF

The presented results do not indicate changes in cardiac energetics (PCr/ATP) after treatment with empagliflozin in nonischemic, mostly nondiabetic patients with HFrEF and with HFpEF. In HFrEF, the failing heart shows an overall decrease in fatty acid oxidation and an increase of glycolysis.^22^ The negative consequences of these derangements are worsened by a significant (≈30%) reduction of ATP content in cardiomyocytes.^40^ In this energetic environment, although ketone bodies may provide a potential carbon-based substrate, the amount of ATP generated per oxygen molecule is considerably less compared with glucose,^41^ which is supported by findings showing that promoting glucose oxidation leads to functional improvements in a preclinical HF model.^42^ Furthermore, uptake of β-OHB is directly proportional to its serum concentrations;^43^ however, despite higher levels of circulating β-OHB levels with 25 mg versus 10 mg of empagliflozin,^44^ there was no measurable difference in subgroup analyses for HF outcomes in EMPA-REG OUTCOME between the 2 doses.^45^ Thus, SGLT2i-induced mild hepatic ketogenesis in response to glucosuria is unlikely to facilitate a change in PCr/ATP.

Little is known about cardiac energetics and substrate metabolism in HFpEF. It was previously established that HFpEF hearts are energy deprived.^26^ Furthermore, our group recently reported that the severity of diastolic dysfunction mirrors the reduction of PCr/ATP in these patients.^27^ Myocardial substrate use appears to differ in patients with preserved versus reduced ejection fraction, and ketones seem to contribute less to ATP generation in the former.^43^ In keeping with these findings, a recent murine HFpEF model showed that β-OHB is not used as an energetic substrate but rather functions as a second messenger, modifying protein hyperacetylation and inflammation.^46^ Consequently, the thrifty substrate hypothesis, ascribing ketones to optimize myocardial energy metabolism by using them as substrates, would not be sufficient to explain treatment effects in HFpEF.

### Serum Metabolites

Recently, a substudy of DEFINE-HF^47^ analyzed serum metabolites to show that treatment with dapagliflozin in patients with HFrEF (n=121) increased metabolite clusters correlated to ketone and long-chain acylcarnitine use.^48^ The authors argue that this underpins the possibility that ketones can restore metabolic balance in the heart and may thus have valuable effects on mitochondrial function in HFrEF. In our study, we did not detect alterations in metabolites relating to overall energy metabolism and likewise no changes in circulating levels of ketone bodies (β-OHB) after empagliflozin. Circulating levels of free fatty acids remained unchanged after treatment. One important distinction regarding our results is that ischemic HF etiology implies limited oxygen supply, which, in itself, even before the onset of HF, alters substrate use and circulating metabolites.^49^ Importantly, meaningful measurements of PCr/ATP (primary end point) require viable myocardium; therefore, all our patients underwent coronary computed tomography angiography before enrollment to exclude significant coronary artery disease. As a result, our cohorts exclusively comprised patients with nonischemic HFrEF, whereas >50% of patients in DEFINE-HF presented with ischemic HFrEF.^47^ In addition, serum metabolomic profiling can only provide a momentary image of circulating metabolites but does not allow inferences of myocardial influx or metabolite use. A significant problem in HF is the uncoupling of circulating substrate availability (high) and their actual use for energy generation (low).^50^ In keeping with this, results from a randomized controlled trial investigating the metabolic modulator perhexiline in patients with nonischemic HFrEF (n=50) demonstrated a significant increase in PCr/ATP (by 30%) but unaltered metabolite extraction in invasively measured (arterial and coronary sinus) samples, emphasizing the limitations of inferring energetic changes based on changes in circulating metabolites alone.^32^ Interestingly, a very recent study investigating plasma and cardiac tissues from patients with HFrEF and HFpEF supports this argument^51^: Cardiac metabolite patterns, but not plasma metabolites, were able to separate HFpEF from HFrEF patients. Furthermore, circulating metabolites of fatty acid metabolism were elevated in plasma samples, but were markedly reduced in myocardial biopsies, suggesting a mismatch between availability and usage. In another study, patient factors for HFpEF showed improved global longitudinal strain measurements on CMR but no changes in fatty acid uptake in the heart after 6 weeks of dapagliflozin treatment.^52^

### Myocardial Triglycerides and SGLT2i

In both patients with HFrEF and HFpEF, we found a trend for a decrease in MTG. Interestingly, MTG correlates with left ventricular hypertrophy in HFrEF, which supports our exploratory findings of reductions in left ventricular (indexed) mass, cell volume, and ShMOLLi T1 in the HFrEF cohort.^53^ Although the degree of diastolic function was reported to correlate to the amount of MTG in HFpEF,^53^ we did not observe significant reductions in diastolic function parameters, which may be a reflection of the reduced sample size due to the COVID-19 pandemic.

### Myocardial Structure, Function, and Quality of Life

Although exploratory and not sufficiently powered, we observed changes indicating reduced hypertrophy and regression of cell volume in the HFrEF cohort. These findings are in keeping with a recent meta-analysis of imaging trials assessing empagliflozin or dapagliflozin in patients with type 2 diabetes and/or HFrEF.^54^ We did not find any meaningful changes in LVEF, which is corroborated by the majority of other CMR trials investigating SGLT2i.^54^ Left ventricular function is closely coupled to myocardial energy metabolism, thus, the lack of functional improvement would not precipitate improvements in PCr/ATP.

**Figure 4.**
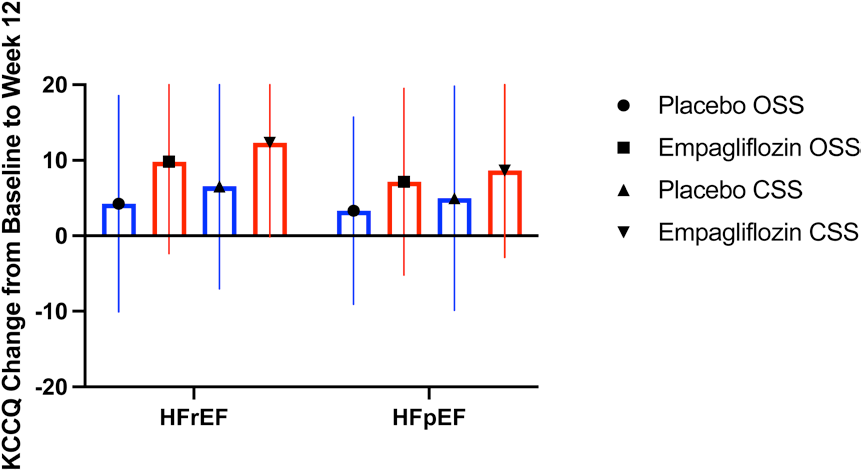
Changes in Kansas City Cardiomyopathy Questionnaire (baseline to week 12). Boxplot showing unadjusted mean change from baseline to week 12 in the overall summary score (OSS) and clinical summary score (CSS) of the Kansas City Cardiomyopathy Questionnaire for the placebo (blue) and empagliflozin (red) groups in patients with heart failure with reduced ejection fraction (HFrEF) and heart failure with preserved ejection fraction (HFpEF). Whiskers indicate SD.

Finally, patients in both treatment groups (empagliflozin and placebo) and both cohorts (HFrEF and HFpEF) showed a trend toward improvements in quality of life (Kansas City Cardiomyopathy Questionnaire), although these were numerically greater in the empagliflozin arm (Figure 4). This is in keeping with previously published results in which an improvement in quality of life has been shown for dapagliflozin and empagliflozin alike.^47,55^ However, this was not paralleled by changes in exercise ability (CPET), which might require longer treatment exposure to empagliflozin (frequently 6 months in other trials) to manifest.^56^

### Limitations

Despite using gold-standard CMR techniques and assessing cardiac as well as whole-body metabolism, our study has several possible limitations. As this is a mechanistic trial, the relatively small sample size, lower-than-anticipated number of patients with type 2 diabetes, and limited ethnic variety in our study may limit the generalizability and comparability of our findings. Patients were only recruited from a single center and investigated twice (before and after treatment). Due to the technical characteristics of our investigation for the primary end point (ie, PCr/ATP), our study is limited to a nonischemic etiology of HF.

We assessed a limited treatment interval of 3 months using a single SGLT2i with one strength (10 mg of empagliflozin); it is unknown whether longer treatment periods, a different SGLT2i, or a higher dose may have led to dissimilar results. Finally, 13 patients (18.1 %) in our HFpEF cohort were excluded from the PPS because of COVID-19 lockdown restrictions. which may have limited the efficacy findings in this cohort.

### Conclusions

EMPA-VISION is the first trial to assess energetics and metabolism after treatment with empagliflozin in patients with HFrEF and HFpEF, respectively. Treatment with empagliflozin (10 mg once daily for a period of 3 months) did not lead to measurable improvements in cardiac energetics (ie, PCr/ATP) at rest or during dobutamine stress. Equally, no changes in a targeted serum metabolomic assay or levels of ketone bodies were observed in either cohort. Thus, our findings could not confirm the thrifty fuel hypothesis presumed to be responsible for the salutary effects observed with SGLT2i in HF. Further research in larger HF cohorts with distinct cardiometabolic phenotypes (eg, those with obesity and diabetes) may be required to assess potentially promising targets possibly affected by treatment with SGLT2i.

## Article Information

### Acknowledgments

The authors express their gratitude toward the Oxford cardiovascular magnetic resonance nursing team, specifically Judith DeLos Santos, Catherine Krasopoulos, Marion Galley, and Claudia Nunes; and the diabetes trials unit team, particularly Irene Kennedy, for her organization skills. The authors also thank the team of the computed tomography suite at the Manor Hospital Oxford as well as all patients who participated in this trial. Drs Holman and Neubauer are Emeritus National Institute for Health Research senior investigators. The views expressed are those of the author(s) and not necessarily those of the National Health Service, National Institute for Health and Care Research, or Department of Health.

### Sources of Funding

Boehringer Ingelheim is the sponsor of the EMPA-VISION study and was involved in early stages of its study design. Boehringer Ingelheim employees (Drs Lee and Massey) also supported preparation of this manuscript. Dr Neubauer acknowledges support from the Oxford British Heart Foundation Centre of Research Excellence. Drs Holman and Neubauer were supported by the Oxford National Institute for Health Research Biomedical Research Centre. Drs Rodgers and Valkovič are funded by Sir Henry Dale Fellowships from the Wellcome Trust and the Royal Society [098436/Z/12/B and 221805/Z/20/Z, respectively]. Dr Valkovič also gratefully acknowledges support of the Slovak Grant Agencies VEGA (Vedecká grantová agentúra) [2/0003/20] and APVV (Slovak Research and Development Agency) [No. 19–0032]. Dr Miller acknowledges support from the Novo Foundation (NNF21OC0068683).

### Disclosures

Dr Hundertmark was supported for this work by an industrial grant provided by Boehringer Ingelheim. Dr Holman reports research support from AstraZeneca, Bayer and Merck Sharp & Dohme, and personal fees from Anji Pharmaceuticals, AstraZeneca, Novartis and Novo Nordisk unrelated to this work. Dr Lee was an employee of Boehringer Ingelheim International GmBH (Ingelheim, Germany) at the time of the study. Dr Rodgers reports funding support by a Sir Henry Dale Fellowship from the Wellcome Trust and the Royal Society. Dr Valkovič reports funding support from the Slovak Grant Agencies. Dr Mahmod reports financial support from Boehringer Ingelheim. Dr Neubauer reports financial support from Boehringer Ingelheim and the Oxford National Institute for Health Research Biomedical Research Centre. The other authors report no conflicts.

### Supplemental Material

Figures S1–S2

Tables S1–S3

Trial Statistical Analysis Plan

## Supplementary Material
