# Supplementary material for: Assessment of Cardiac Energy Metabolism, Function, and Physiology in Patients With Heart Failure Taking Empagliflozin: The Randomized, Controlled EMPA-VISION Trial
Source: Circulation. 2023 Apr 18;147(22):1654–69. doi: 10.1161/CIRCULATIONAHA.122.062021 (PMC10212585; doi:10.1161/CIRCULATIONAHA.122.062021)
Supplement: Supplementary file 1 [file cir-147-1654-s001.pdf]

SUPPLEMENTAL MATERIAL

Supplemental Figure S1

Absolute changes (week 12 – baseline) in resting and dobutamine stress PCr/ATP

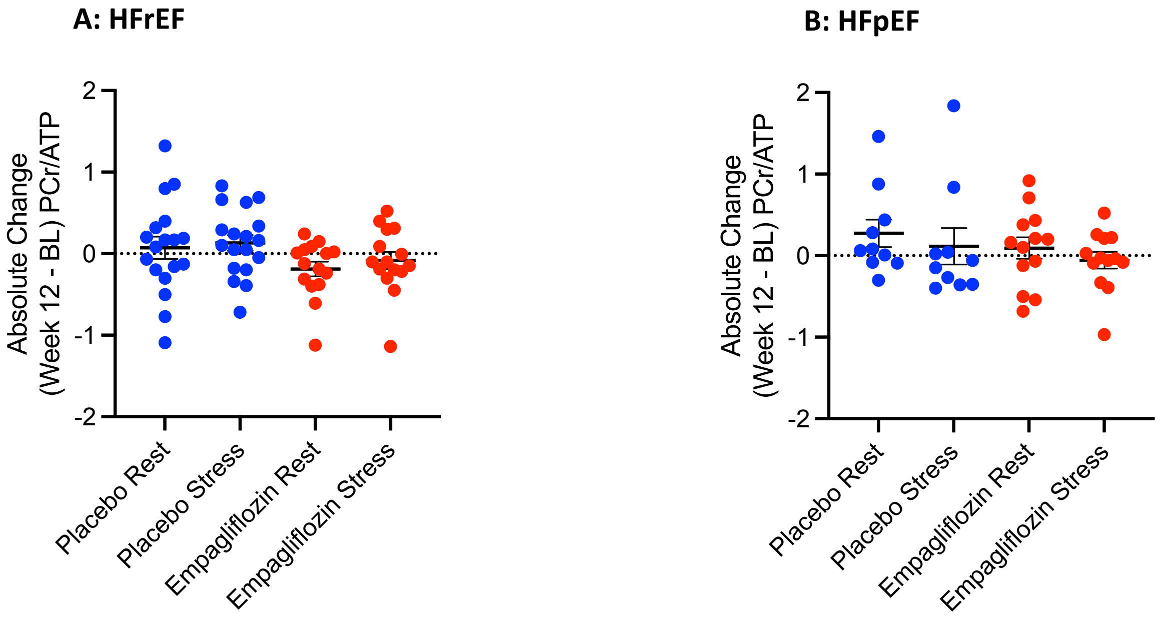

Supplemental Figure 1. Absolute Changes in PCr/ATP defined by ^{31}P-MRS *A: HFrEF (left panel), B: HFpEF (right panel). Scatter plots (including mean and SEM) calculated by subtracting the baseline values from the week 12 values (i.e. week 12 – baseline) to elicit the absolute changes in this measure. Data points in blue represents patients treated with placebo while red data points represent patients treated with empagliflozin (10mg OD) at rest ('Rest') or during dobutamine stress (65 % of age-maximal heart rate; 'Stress'), respectively.*

Supplemental Figure S2

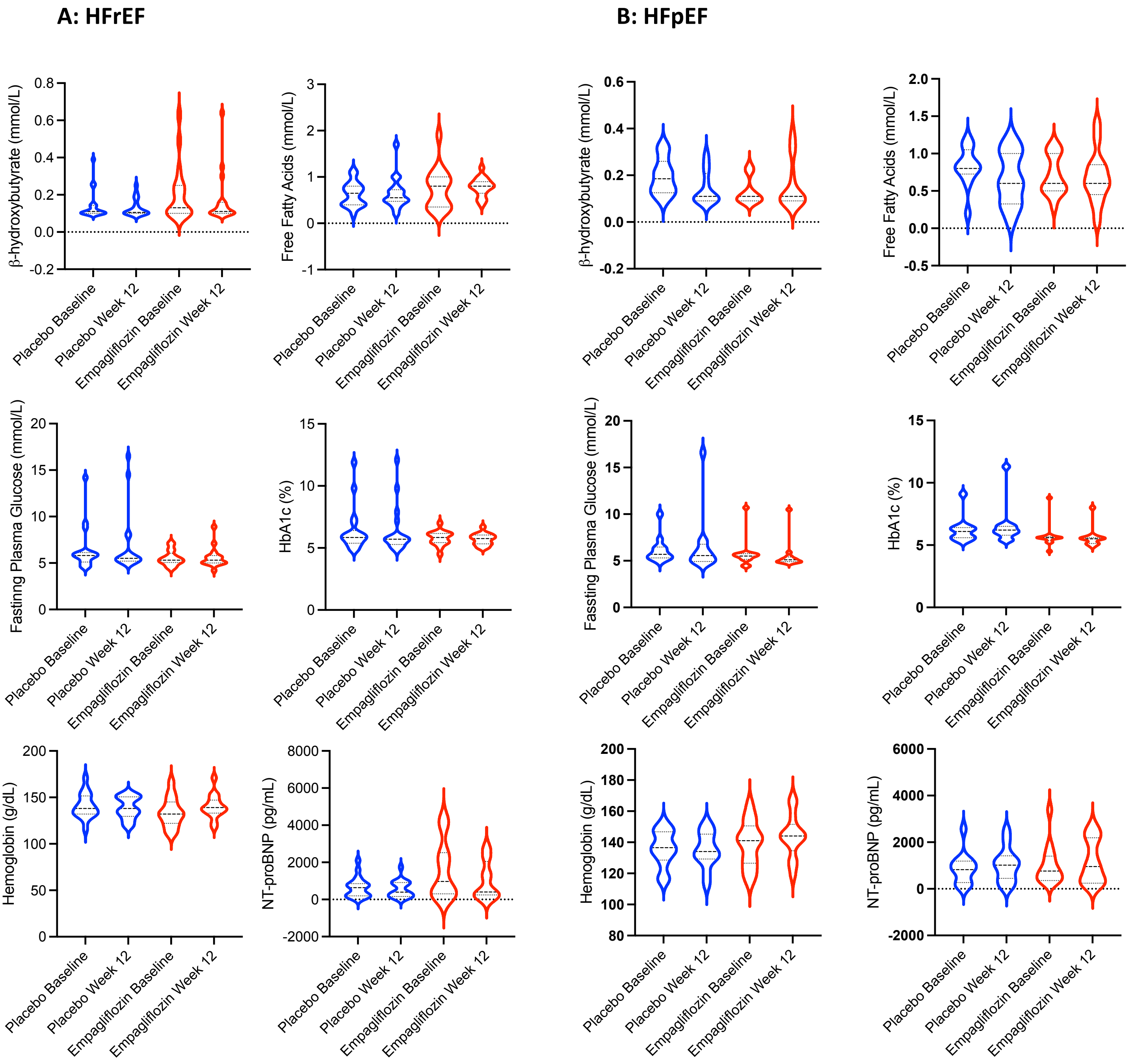

Baseline and post-treatment (week 12) values of selected biomarkers with empagliflozin (red) and placebo (blue) in patients with heart failure and reduced ejection fraction (A; HFrEF) and heart failure with preserved ejection fraction (B; HFpEF), respectively. dL=deciliter; g=gram; HbA1c=Glycated hemoglobin A1c; L=liter; mmol=millimole; NT-proBNP=n-terminal pro B-type natriuretic peptide

Supplemental Table S1.

Detailed in- and exclusion criteria for EMPA-VISION

Inclusion Criteria EMPA-VISION

• CHF ≥ 3 months
• NYHA II-IV at screening
• BMI < 40 kg/m^2
• Age ≥ 18 years
• Written informed consent

HFrEF	HFpEF
• LVEF < 40% (measured by Echocardiogram)<br>• NT-proBNP (> 125 pg/mL) no AF<br>• NT-proBNP (> 600 pg/mL) if diagnosed AF<br>• Stable optimal medical therapy	• LVEF ≥ 50% (measured by Echocardiogram)<br>• Structural left heart disease (LAVI ≥ 34 ml/m^2; LVMI ≥ 115 g/m^2 for males and ≥ 95 g/m^2 for females)<br>• NT-proBNP (>125 pg/mL) no AF<br>• NT-proBNP (>600 pg/mL) if diagnosed AF<br>• Optimal medical therapy<br>• Stabe dose of diuretics for > 1 week

Exclusion Criteria EMPA-VISION

•Stroke or TIA < 6 months
•Scars or non-viable myocardium in the interventricular septum, unstable angina pectoris due to CAD, major CV surgery (investigator opinion)
•Any contraindication for CMR, CPET, dobutamine stress test
•Heart transplant recipient or listed for heart transplant
•Cardiomyopathy based on infiltrative diseases (e.g. amyloidosis), accumulation diseases (e.g. Haemochromatosis, Fabry's disease), muscular dystrophies, cardiomyopathy with reversible causes (e.g. stress cardiomyopathy), hypertrophic obstructive cardiomyopathy or known pericardial constriction
•Moderate to severe uncorrected primary valvular heart disease
•Acute decompensated HF (exacerbation of CHF) requiring i.v. diuretics, i.v. inotropes or i.v. vasodilators, or LVAD or hospitalisation
•SBP ≥ 180 mmHg at screening. If SBP > 150 mmHg and < 180mmHg at screening on antihypertensive triple therapy
•Symptomatic hypotension and/or a SBP < 100 mmHg at screening
•Uncontrolled AF
•Untreated ventricular arrhythmia with syncope documented within the 3 months prior to informed consent in patients without ICD
•Diagnosis of cardiomyopathy induced by chemotherapy or peripartum < 12 months prior to informed consent
•Symptomatic bradycardia or second or third-degree heart block in need of a pacemaker after adjusting beta-blocker therapy or any other negative inotropic agents
•Significant chronic pulmonary disease
•Indication of liver disease, defined by serum levels of either ALT, AST, or AP above 3 x upper limit of normal
•Impaired renal function (Creatinine Clearance < 30 mL/min and/or dialysis)
•Haemoglobin < 10 g/dL
•T1DM
•History of ketoacidosis
•Major surgery < 3 months prior or scheduled within trial
•GI surgery or significant GI disorder
•Active or suspected malignancy or history of malignancy within 2 years prior to informed consent

•Any other disease than HF with a life expectancy of < 1 year<br>•Any drug considered likely to interfere with the safe conduct of the trial<br>•Requirement for treatment with empagliflozin<br>•Treatment with any SGLT2i or combined SGLT1- and SGLT2i<br>•Currently enrolled in another investigational device or drug study, or < 30 days between randomisation and ending the other investigational trial<br>•Known allergy or hypersensitivity to empagliflozin or other SGLT2-i<br>•Chronic alcohol or drug abuse<br>•Women who are pregnant, breastfeeding, or who plan to become pregnant while in the trial

Supplemental Table S2

Visit Schedule and investigations conducted on each respective visit.

**Trial Period**	**Screening**	**Treatment**			**Follow-up***
**Visit**	**1**	**2****	**3**	**4****	**5**
**Timing**	**1-21 days prior to randomisation**	**Day 1**	**Day 15** ±1 day	**Day 84/ EOT** ±4 days	**7-14 days after last dose**
Informed consent	X				
Demographics, alcohol and smoking history	X				
NYHA classification	X			X	
Cardiac computed tomography (CT) scan	X				
Echocardiogram (ECHO)	X[1]	X[2]	X	X	
Safety laboratory tests (blood)	X	X[2]		X	
Safety laboratory tests (urine)			X[3]		
Blood sampling for NT-proBNP asessment	X				
Blood sampling for biomarker assessment, including NT-proBNP		X		X	
Blood sampling for metabolomic analysis		X		X	
12 lead-electrocardiogram (ECG)	X[1]	X[2]		X	
Vital signs	X	X[2]	X	X	
Height	X				
Weight	X	X[2]	X	X	
Pregnancy test[4]	X	X	X	X	
Review of in-/exclusion criteria	X	X			
Medical history	X				
Physical examination	X			X	
Randomisation		X			
Dispense trial medication		X			
Administer trial medication		Continuous daily dosing[5]			
Medication compliance check			X	X	
Cardio-pulmonary Exercise test (CPET)		X[6]		X[6]	
6 Minute Walk Test (6MWT)		X[6]		X[6]	
Cardiac Magnetic Resonance (CMR)		X		X	
Patient reported outcomes (questionnaires)		X		X	
Adverse events	X	X	X	X	X
Concomitant therapy	X	X	X	X	X
Telephone call to patient					X
Completion of patient participation					X

- The follow-up visit must be completed for all randomised patients, including those who withdraw prematurely), unless the patient has withdrawn consent to follow-up. The follow-up visit is expected to be performed via a telephone call, but a clinic visit may be performed at the discretion of the investigator.

** Patients must be fasting at Visits 2 and 4.

1 If echocardiogram and/or electrocardiogram is/are performed as part of standard care within 3 months prior to informed consent, and written results are available, the assessment does not need to be repeated during the screening visit and the existing scan(s) can be used to assess eligibility.

2 The following assessments do not need to be repeated at visit 2 if performed within the previous 7 days; Safety laboratory tests (blood), Echocardiogram, Electrocardiogram, Vital signs, Weight.

3 At Visit 3 only a urine test for ketone bodies is required.

4 For women of childbearing potential either a urine or serum pregnancy test is required.

5 Daily dosing should continue until the last dose is taken on the day of Visit 4.

6 CPET and 6MWT may be performed 1 day before or after the visit (but must be performed after randomisation and before first dose at Visit 2).

Supplement Table S3.

Overall summary of adverse events (AE) according to treatment group (placebo vs. empagliflozin).

Overall summary of adverse events according to treatment (placebo vs. empagliflozin)

	Placebo<br>*N (%)*	*Empa 10 mg*<br>*N (%)*
Number of patients	36 (100.0)	35 (100.0)
Patients with any AEs	19 (35.3)	17 (48.6)
Patients with severe AEs	4 (11.1)	0
Patient with investigator defined trial drug-related AEs	4 (11.1)	5 (14.3)
Patients with investigator defined dobutamine-related	0	0
Patients with AEs leading to discontinuation of trial drug	2 (5.6)	1 (2.9)
Patients with serious AEs	7 (19.4)	1 (2.9)
Results in death	0	0
Is life threatening	1 (2.8)	0
Persistence of significant disability/incapacity	0	0
Requires or prolongs hospitalisation	4 (11.1)	1 (2.9)
Congenital anomaly of birth defect	0	0
Other medically important serious event	7 (19.4)	1 (2.9)

Trial Statistical Analysis Plan

Study Objectives

The objective of this trial is to assess the effect of empagliflozin on cardiac physiology and metabolism aiming to provide a scientific explanation of the underlying mechanism by which empagliflozin improves HF related outcomes in patients with chronic heart failure.

Primary Objectives

The primary endpoint is the change from baseline (Randomisation) to week 12 (Day 84) in the PCr / ATP ratio in the resting state measured by ^{31}P MRS.

Secondary Objectives

There are no secondary endpoints defined in this trial.

Exploratory Objectives

The exploratory objective of this trial is to document the effects of empagliflozin on various parameters affected by heart failure and to investigate how these relate to the cardiac effects of empagliflozin.

The following exploratory endpoints will be assessed:

- Changes from baseline to week 12 in functional capacity assessed by Cardiopulmonary Exercise Testing (CPET) and 6 Minute Walk Test (6MWT)
- Changes from baseline to week 12 in cardiac structure and function measured by magnetic resonance imaging (MRI) and echocardiogram
- Changes from baseline to week 12 in myocardial fat composition measured by proton MRS
- Changes from baseline to week 12 in short-term quality of life (QoL) as measured by the EQ-5D and KCCQ questionnaires
- Change from baseline to week 12 in ΔPCr/ATP ratio measured by ^{31}P MRS at rest and under dobutamine stress
- Change from baseline to week 12 in PCr/ATP ratio during stress test measured by ^{31}P MRS
- Change from baseline to week 12 in myocardial function during stress as measured by CMR cine imaging
- Change from baseline to week 12 in resting myocardial blood flow as measured by CMR rest perfusion
- Change from baseline to week 12 in extracellular volume as measured by CMR native and post contrast T1 mapping
- Change from baseline to week 12 in left atrial volume and emptying (left atrial emptying function (LAEF) by CMR
- Change from baseline to week 12 in biomarker assessments

Overall trial design and plans

This randomised, double-blind, placebo-controlled trial investigates the effects of empagliflozin on cardiac metabolism and function in heart failure patients. Empagliflozin will be compared to placebo as add-on to standard of care treatment in heart failure patients. The effects of empagliflozin will be assessed independently in two separate cohorts of patients; those with reduced ejection fraction (rEF) and those with preserved ejection fraction (pEF), but the randomised treatment and trial assessments for both cohorts are identical.

Figure 1. Trial Design

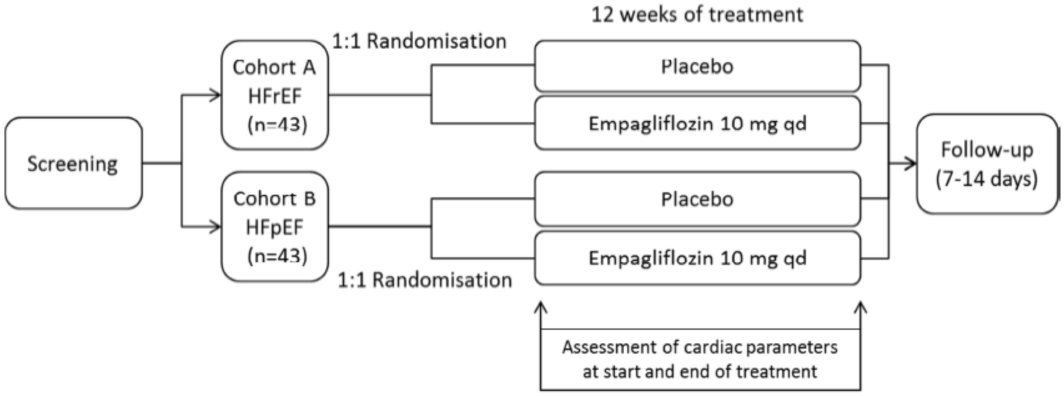

Clinical Event Committee for Diabetic ketoacidosis (DKA)

An independent external committee (CEC) will be established to adjudicate centrally and in a blinded fashion events suspected of DKA. The CEC will evaluate whether pre-specified criteria for adjudication endpoints are met.

For any events that qualify for adjudication, study sites will be asked to provide clinical documentation such as laboratory values, discharge summaries etc. to support the external event adjudication. The tasks and responsibilities of the CEC will be specified in a charter. The CEC will maintain the adjudication results in writing.

Hepatic external adjudication

Certain hepatic events will be adjudicated by external independent experts for severity and a causal relationship with the trial medication in a blinded fashion. The events which will be reviewed are defined in a charter for hepatic events. Events may either be defined by abnormal laboratory values and/or relevant adverse events or both. For example, assessments will be made for hepatic injury events, including liver enzyme elevations.

For qualifying events, relevant source documents generated from any medical evaluations of these events will be requested, including laboratory values, histological analysis, and results of ultrasound, CT, MRI, scintigraphy, hospital discharge letters, and medical reports from other physicians. All evaluations will be performed in a blinded fashion. The assessments will be analysed based on empagliflozin data combined from multiple trials (i.e. on project level).

Changes to the Planned Analysis

The main statistical model defined in the protocol does not include baseline response as a covariate, as baseline response is usually predictive of response the following changes to the protocol have been introduced:

- A sensitivity analysis of the primary efficacy endpoint has been included that includes baseline in an analysis of covariance (ANCOVA) model. Note the main analysis of the primary efficacy endpoint remains as defined in the protocol (i.e. without the addition of baseline as a covariate).
- The analysis of variance (ANOVA) model will be replaced by an ANCOVA model for all applicable exploratory endpoints in order to fit baseline response as a covariate.

Endpoints

Absolute change (Δ): will be defined as the value obtained at Week 12 minus value obtained at Baseline [Δ = Week 12 (Value) -Baseline (Value)]. Positive value represents an increase from baseline. Negative value represents a decrease from Baseline.

1 Definition and Derivation of Primary Endpoint

The primary endpoint is the change from the baseline to week 12 in PCr/ATP ratio in resting state measured by ^{31}PMRS after treatment with Empagliflozin or matching placebo.

2 Primary Hypothesis under Investigation

The null hypothesis is that for patients in each of the HFrEF and HFpEF cohorts, the change from the baseline to week 12 in PCr/ATP ratio in resting state measured by ^{31}P Magnetic Resonance Spectroscopy (MRS) after treatment with Empagliflozin is not different from matching placebo. The alternative hypothesis is that the change is different.

3 Handling of Outliers and Missing Data

For the primary outcome, patients who do not have a measurement taken at baseline and week 12 will be excluded from the primary analysis. For primary analyses, missing efficacy data will not be imputed.

Any outliers will be identified and documented prior to the database lock and unblinding. Cases that are visually "extreme" will be assessed for possible influence on the results and conclusions by comparing results from analyses with or without outliers. Any discrepant results from the two analyses will be reported and discussed in the CTR and publication manuscripts.

Secondary Endpoints

1 Definitions and Derivations of Secondary Endpoints

No secondary endpoints are defined for this trial.

2 Secondary Hypotheses under Investigation

No secondary hypotheses are defined for this trial.

Exploratory Endpoints

1 Definitions and Derivations of Exploratory Endpoints

The following exploratory endpoints will be assessed to investigate changes from baseline to week 12 after treatment with Empagliflozin or matching placebo:

Changes from baseline to week 12 in functional capacity assessed by Cardiopulmonary Exercise Testing (CPET). Parameters given as:

- Forced vital capacity (FVC)
- Forced expiratory volume at 1s (FEV1)
- Peak VO_2
- Ventilation/carbon dioxide (VE/VCO_2) slope
- Ventilatory threshold
- Respiratory Exchange Ratio (RER)
- Lactate
- SpO_2
- Systolic BP
- Diastolic BP
- Heart rate
- Exercise time
- Maximal workload
- Rating of perceived exertion (RPE) score

Changes from baseline to week 12 in functional capacity assessed by 6 Minute Walk Test (6MWT). Parameters given as:

- Total distance covered
- Borg score dyspnoea
- Borg score fatigue
- Heart rate
- SpO_2

Changes from baseline to week 12 in parameters measured by cardiac magnetic resonance imaging (CMR) given as:

- PCr/ATP (rest – stress)
- PCr/ATP under dobutamine stress
- Myocardial fat composition
- Resting myocardial blood flow (via CMR rest perfusion)
- Myocardial function parameters during rest and stress (via cine imaging): Stroke volume during REST
- Ejection fraction during REST
- Peak systolic circumferential strain during REST
- Peak systolic longitudinal strain during REST
- Peak systolic radial strain during REST
- Peak longitudinal diastolic strain rate during REST
- Peak circumferential diastolic strain rate during REST
- Stroke volume during STRESS
- Ejection fraction during STRESS
- Peak systolic circumferential strain during STRESS
- Peak systolic longitudinal strain during STRESS
- Peak systolic radial strain during STRESS
- Peak longitudinal diastolic strain rate during STRESS
- Peak circumferential diastolic strain rate during STRESS

Cardiac structure and function parameters (via MRI):

- Peak systolic circumferential strain
- Peak systolic longitudinal strain
- Peak systolic radial strain
- Peak circumferential diastolic strain rate
- Peak longitudinal diastolic strain rate

- Torsion
- Stroke volume
- Ejection fraction
- Left ventricular (LV) mass
- LV mass index

Extracellular volume (via CMR native and post contract T1 mapping):

- Native T1 (average)
- Native T1 (threshold)
- Native T1 (lesions)
- Presence of non-ischaemic pattern of LGE (fibrosis)
- Quantification of non-ischaemic pattern of LGE (fibrosis)

Left atrial volume and emptying:

- LV end diastolic volume during rest
- LV end systolic volume during rest
- Left atrial volume during rest (diastolic volume – systolic volume)
- LV end diastolic volume during stress
- LV end systolic volume during stress
- Left atrial volume during stress (diastolic volume – systolic volume)
- LV end diastolic volume
- LV end systolic volume
- Left atrial volume (diastolic volume – systolic volume)

Changes from baseline to week 12 in cardiac structure and function measured via echocardiogram. Parameters given as:

- Left Ventricular Ejection Fraction (LVEF)
- LV end-diastolic volume
- LV end-systolic volume
- LV mass index
- Septal e-velocity
- Lateral e-velocity
- E/e ratio
- Left atrial volume index
- Chamber size
- Chamber thickness
- Chamber wall motion
- Haemodynamic status (cardiac output)
- Valves status

Changes from baseline to week 12 in short-term QoL as measured by the EQ-5D, for the UK utility score and the EQ-VAS score. The utility score will be calculated using UK preference weights as detailed in Section 7.2.2.

Changes from baseline to week 12 in short-term QoL as measured by KCCQ questionnaires for each of the 10 summary scores, as defined and derived in Section 7.2.1.

Change from baseline to week 12 in biomarker assessments. Parameters given as:

- Aceto-acetate
- Aldosterone
- Angiotensin II
- Beta-hydroxybutyrate
- Brain natriuretic peptide (BNP)
- Erythropoetin
- Fasting plasma glucose
- Free fatty acids (FFA)
- HbA1c
- NT-proBNP
- Renin Activity
- Direct Renin Concentration

Handling of Missing Data

None or negligible amount of missing data values is expected for the identified exploratory variables. In a situation where there are missing data values for any variables, only a complete case (data without imputation using randomised set) analysis will be reported in the CTR.

Analysis Sets/Populations

The following analysis sets are defined in this trial.

1 Per-protocol Set (PPS)

The primary endpoint analysis will be performed using the per protocol (PP) set of patients having a valid PCr/ATP ratio measurements available at baseline and week 12, and not having an important protocol violation relevant to the primary endpoint. Protocol violations will be reviewed by the study team before the database lock to determine which violations disqualify a patient from the Per-Protocol analysis.

2 Randomised Set (RS)

This set will include all subjects who are randomised to study treatment. The RS set will be employed for a sensitivity analysis to check for an unbiased estimation of the primary endpoint result if a substantial number of protocol violations resulted in a reduced sample size used for the primary endpoint estimates. The analysis of exploratory endpoints will be performed on the RS of patients with available data.

3 Treated set (TS)

All randomised and treated patients will be included in the safety analysis and safety summaries will be presented by actual treatment received.

General Issues for Statistical Analysis

Patients randomised into the incorrect stratum, cohort (HFrEF or HFpEF) and/or history of diabetes (Yes or No) will be analysed according to the stratum that they should have been randomised into.

The summary statistics and statistical estimates (effect sizes) will be presented to one decimal place greater than the raw data collected. All p-values will be rounded to 4 decimal places. All estimates of treatment effects will be presented with corresponding 95% confidence intervals.

1 Analysis Software

All statistical analyses will be conducted using SAS 9.4, software. All the statistical analyses outputs/results will be reviewed and validated by an independent statisticians.

2 Multiplicity, Multiple Comparisons and Interim Analyses

If recruitment is different between the cohorts such that one cohort's last patient out visit is considerably earlier than the last patient out of the other cohort, an interim analysis may be planned in order to report the data the completed cohort. For this interim analysis only the completed cohort will be unblinded.

A single hypothesis is being tested for each cohort for a single primary efficacy endpoint. The analysis of all other endpoints will be considered exploratory in nature with nominal p-values. All p-values will be two-sided at an alpha-level of 0.05.

3 Planned Subgroup Analyses

Subgroup analyses will be performed for the primary endpoint only, to identify changes in the treatment effect in different predefined subgroups (Baseline eGFR (<60, >=60), History of diabetes and History of atrial fibrillation).

Disposition of Patients

All patients in the screened set (signed informed consent) will be included in the summary of patient disposition. Frequencies and percentages of the total number of patients screened, randomised, treated, prematurely discontinued treatment (along with reason for discontinuation) and status at trial completion will be summarised in tabular forms for each cohort and for both cohorts overall.

A summary of the frequency and percentage of patients by treatment group will be produced for the randomised, treated and per-protocol analysis sets. Summaries by cohort and overall will be produced.

Demographic and Baseline Characteristics

The screening demographic and the clinical characteristics will be tabulated and summarised by the treatment group. The demographic and baseline data will include gender, age, race, weight, height, SBP, DBP, T2DM, HAF, alcohol use, smoking, heart rate, vital signs medical history, and other cardiac parameters (EF, BNP) measured at screening.

Primary Endpoint Analysis

1 Primary Efficacy Analysis

The primary efficacy endpoint of change from baseline to week 12 in PCr/ATP will be analysed for each cohort (HFrEF and HFpEF) separately, to test the hypothesis detailed in Section 2.1.2.

The analytical procedure will include a descriptive summary and a formal (inferential) statistical summary, ANOVA, for the primary endpoint hypothesis testing. Plots of individual patient changes from baseline to week 12 will be produced by treatment group and cohort, including the mean change also.

2 Formal analysis

The formal statistical analysis will employ an Analysis of Variance (ANOVA) model to test the primary hypothesis for each individual cohort using the following for the model inputs:

- Response (outcome) variable is defined as:
  The change (PCr/ATP absolute change) from baseline to week 12 is calculated for each patient by subtracting each patient's baseline PCr/ATP measurement from the 12 weeks measurement.

- Factors:
  Treatment (Empagliflozin vs. Placebo), History of diabetes (Yes/No) and History of atrial fibrillation *(Yes/No)*

The null hypothesis of no difference in change between the treatment and placebo groups will be tested by an ANOVA on the PCr/ATP ratio absolute change using treatment (empagliflozin vs. placebo), history of diabetes (yes vs, no) and history of atrial fibrillation (yes vs no) as fixed effects. If the treatment main effect is significant then we reject the null hypothesis.

The formal model will be specified in SAS as follows (equivalent to ANOVA):

PROC MIXED data=values cl method=reml covtest;

CLASS af diab trt;

MODEL endpt = af diab trt / ddfm=kr solution;

LSMEANS trt / cl diff om alpha=**0.05**;

RUN;

Though the primary endpoint is regarded to generally be normally distributed some model checking will be performed. The model adequacy assessment will be based on the normality of residuals, homogeneity of variance between groups and homoscedasticity. Any violation of the model assumptions will be investigated and ratified accordingly and will be reported in the clinical trial report.

3 Sensitivity and Exploratory Analyses

The following sensitivity analyses are planned:

- A repeat of the primary analysis model but based on the RS of patients
- A repeat of the primary analysis model but including baseline PCr/ATP as a covariate in the model (i.e. an ANCOVA), for the PPS

The SAS code for the ANCOVA model will be as follows:

PROC MIXED data=values cl method=reml covtest;

CLASS af diab trt;

MODEL endpt = baseline af diab trt / ddfm=kr solution;

LSMEANS trt / cl diff om alpha=**0.05**;

RUN;

4 Subgroup Analyses

Subgroup analyses will be performed to assess the homogeneity of treatment effect on changes in the PCr/ATP ratio in different subgroups (eGFR (<60 v >=60), diabetes and atrial fibrillation subgroups). The same ANOVA model as used for the primary endpoint will be employed with the addition of subgroup term (if not already fitted) and the treatment by subgroup interaction term. Treatment effects within each subgroup level will also be estimated using the same model but with the interaction and subgroup terms removed. A Forest plot of the overall treatment effect (with 95% CIs) along with treatment effects within each subgroup level will also be produced.

5 Secondary Endpoint Analyses

Not applicable as no secondary endpoints defined for the study.

6 Exploratory Analyses

A full list and derivation of the exploratory endpoints for this trial is provided above. The endpoints will either just have a descriptive summary performed or be analysed via ANCOVA. All summaries will be produced by each of the cohorts and performed on the RS using all available data.

The ANCOVA model will include the fixed effects of treatment, eGFR group, history of diabetes and history of atrial fibrillation along with the baseline measure of the endpoint fitted as a covariate. The following exploratory endpoints will have an ANCOVA performed all other endpoints will only be descriptively summarised:

- Change from baseline to week 12 in CPET: all parameters
- Change from baseline to week 12 in 6MWT: distance covered, HR and SpO2
- Change from baseline to week 12 in PCr/ATP (rest - stress) and stress PCr/ATP
- Change from baseline to week 12 in myocardial fat composition
- Change from baseline to week 12 in myocardial function parameters during rest and stress
- Change from baseline to week 12 in MRI cardiac structure and function parameters
- Change from baseline to week 12 in myocardial blood flow (via rest perfusion)
- Change from baseline to week 12 in T1 mapping parameters
- Change from baseline to week 12 in left atrial parameters

All ANCOVA estimates are for exploratory purposes and the resulting p-values and/or confidence intervals will be interpreted with caution.

Safety and Tolerability Analysis

All safety and tolerability summaries will be produced on the treated set of patients.

1 Drug Exposure

Empagliflozin 10 mg for 12 weeks, see for the drug exposure and accountability plans throughout the trial. Both empagliflozin and placebo exposure will be tabulated, duration of exposure will be calculated from the day of first exposure to the day of last exposure, ignoring any missed doses between. Cumulative dose (mg) will be summarized using descriptive statistics, where cumulative

dose is the actual dose taken summed across all visits, missed days will be counted as 0 mg. The number of missed doses will also be tabulated.

2 Adverse Events and Tolerability

Statistical analysis and reporting of adverse events will concentrate on treatment-emergent adverse events, i.e., all adverse events occurring between the start of treatment and end of the residual effect period, 7 days after the last dose of medication. Adverse events that start before first drug intake and deteriorate during the treatment period will also be considered as ‘treatment-emergent’. All AEs will be listed, according to whether the AE start date occurred during the screening period, treatment period, or during the follow-up period.

The safety analyses will be purely descriptive statistics *(number and percentage, n (%))* and will be based on the current version of Dictionary of Drug Regulation (MedDRA) coding system and display in tables and data listings using SOC and preferred term. The analyses will be provided for each cohort and also pooled across all cohorts. No hypothesis testing is planned. An overall summary of AEs will be presented, in addition the following tables will be produced by MedDRA SOC and preferred term:

- All AEs
- AEs by severity
- Serious AEs
- Drug-related AEs
- AEs leading to treatment discontinuation

In addition, a summary of all AEs by investigator-defined category and MedDRA preferred term will be produced, the following categories will be included:

- Hypoglycaemic event
- Genital infection
- Acute pyelonephritis
- Sepsis
- Urinary tract infection
- Bone fracture
- Hepatic injury
- Ketoacidosis (metabolic acidosis, ketoacidosis, diabetic ketoacidosis)
- Decreased renal function
- Other

Further details on the specific AEs of hypoglycaemia, genital infections, acute pyelonephritis, sepsis, bone fractures and ketoacidosis will be listed. Additional summaries of these specific AEs and other specific AEs (e.g. volume depletion) may be produced dependent upon the frequency of occurrence.

3 Laboratory Data

Descriptive statistics (Mean (SD) and Median (IQR)) of the laboratory data will be presented. The presentation will cover data collected at screening or baseline to week 12 (screening to treatment period). Summaries of actual values at baseline and last value on-treatment along with the change from baseline will be tabulated for the individual parameter. Baseline data will be taken as the last available laboratory measurement on or prior to the day of starting study treatment. The individual

patient values profile over time will be plotted. The marginal means of individual parameter will also be included in these plots. Laboratory values will be compared to their reference ranges at baseline and the last measurement on-treatment. Frequency tables will summarise the number of patients with potentially clinically significant abnormalities (as defined by BI project standards). Laboratory measurements taken up to 3 days after the last administration of randomised study treatment will be considered as on-treatment.

4 Vital Signs, Electrocardiogram, and Other Safety Assessments

Vital signs, ECG and other safety assessments will be summarised descriptively.

Data Handling Conventions

5 Data Quality Assurance and Monitoring

The data monitoring and quality assurance procedures for the study can be provided upon reasonable request with the Study's Principal Investigator.
